# An analysis of factors related to the development of in-stent restenosis after percutaneous coronary intervention: Retraction

**DOI:** 10.1097/MD.0000000000019412

**Published:** 2020-02-21

The article, “An analysis of factors related to the development of in-stent restenosis after percutaneous coronary intervention”,^[1]^ which appears in Volume 99, Issue 5 of *Medicine*, is being retracted at the request of the authors due to concerns over reproducibility, inadequate sample size and bias in the results of the statistical analysis.
